# Functioning of the Masticatory System in Patients with an Alloplastic Total Temporomandibular Joint Prostheses Compared with Healthy Individuals: A Pilot Study

**DOI:** 10.3390/life12122073

**Published:** 2022-12-10

**Authors:** Caroline M. Speksnijder, Nadiya E. A. Mutsaers, Sajjad Walji

**Affiliations:** 1 University Medical Center Utrecht, Department of Oral and Maxillofacial Surgery and Special Dental Care, Utrecht University, 3584 CX Utrecht, The Netherlands; 2 Department of Oral and Maxillofacial Surgery, Jeroen Bosch Hospital, 5223 GZ ‘s-Hertogenbosch, The Netherlands

**Keywords:** mastication, bite force, alloplastic total joint replacement, temporomandibular joint

## Abstract

Background: Most patients with temporomandibular joint (TMJ) issues are successfully treated with nonsurgical methods. However, when end-stage TMJ pathologies occur, invasive management can be required, such as a total TMJ replacement. This cross-sectional pilot study aimed to provide insight into the functioning of the masticatory system, pain, and patient satisfaction in patients treated with a total joint replacement (TJR). Methods: A cross-sectional pilot study was conducted to determine the postoperative clinical results of an alloplastic TJR TMJ. Masticatory performance and also insight into maximum voluntary bite force (MVBF), active and passive maximum mouth opening (aMMO/pMMO), pain, and patient satisfaction were measured. Masticatory performance, MVBF, and aMMO of patients with a TJR TMJ were compared with healthy individuals. Results: Masticatory performance is equal between patients with a TJR TMJ and healthy individuals, but both MVBF and aMMO were significantly smaller in patients with a TJR TMJ. However, patients had almost no pain and were very satisfied with the TJR TMJ treatment. Conclusion: This study revealed that most patients with an alloplastic TJR TMJ were able to function without pain, showed good masticatory performance, and were highly satisfied with their alloplastic TJR TMJ. However, MVBF and aMMO were lower than in healthy individuals.

## 1. Introduction

The temporomandibular joint (TMJ) is a complex articulation in the human body, supporting functions such as mastication and mouth opening [1]. When oral functions are impaired by issues with the TMJ, temporomandibular dysfunction (TMD) can occur [1,2,3]. The prevalence of TMD pain complaints in the Dutch general population ranges between 7.2 and 8.0% and is more reported in women [4,5]. In most cases, patients with TMJ issues are successfully treated with nonsurgical methods, such as physiotherapy, splint therapy, behavioral therapy, and pharmacological treatment [6]. However, between 3 and 5% of the population who seek help for their TMJ issues are not successfully treated with nonsurgical methods and require invasive management [7]. When end-stage TMJ pathologies occur, such as severe osteoarthritis, inflammatory arthrosis, fibrous and bony ankyloses, deformities such as excessive condylar resorption, autoimmune disease, certain congenital disorders, trauma, chronic pain, and multiple failed prior TMJ surgeries [2,8,9], invasive management can be required by a total joint replacement (TJR) of the TMJ [10,11,12]. Important goals of this joint replacement are the optimization of mandibular function, such as mastication, and a reduction in pain [13].

Surgical replacement by an alloplastic TJR TMJ has shown to significantly increase maximum voluntary bite force (MVBF), maximum mouth opening (MMO), and masticatory functioning and reduces pain and diet restriction due to these [2,8,14,15]. To our knowledge, masticatory functioning is only obtained by patient-reported outcomes (PROs) in patients with a TJR TMJ. With such a PRO, the patient’s own perception of mastication can be measured, which is defined as masticatory ability. However, with the mixing ability test (MAT), mastication can be measured objectively, which is defined as masticatory performance. This MAT assesses the comminution of a bolus by a standard number of chewing cycles [16,17,18,19,20]. Measuring masticatory performance will complement the knowledge of masticatory ability in patients with an alloplastic TJR TMJ [20,21]. Therefore, the first aim of this cross-sectional pilot study was to provide insight into masticatory performance, but also insight into MVBF, active MMO (aMMO), passive MMO (pMMO), pain, patient satisfaction related to their alloplastic TJR TMJ, and masticatory ability in patients who received an alloplastic TJR TMJ. The second aim was to compare masticatory performance, MVBF, and aMMO between patients who received an alloplastic TJR TMJ and matched healthy individuals.

## 2. Materials and Methods

A cross-sectional pilot study was conducted to determine the postoperative clinical results of an alloplastic TJR TMJ. Patients who received an alloplastic TJR TMJ during the period January 2013 to April 2018 at the Jeroen Bosch Hospital (‘s-Hertogenbosch; The Netherlands) were contacted for recruitment and examination. In- and exclusion criteria for patients are depicted in Table 1. All patients received a letter by email including information about the study. Within 2 weeks, the patients were contacted by telephone to ask whether they were willing to participate in this study and to provide more information about the study when needed. Patients who were willing to participate were invited to Jeroen Bosch Hospital for screening. Approval was obtained from the Medical Ethics Committee Brabant (file no. NL65072.028.18).

We matched the included patients in this study with healthy individuals on age, gender, and dental state. These healthy individuals had no temporomandibular deficits and were part of a former study in which the mixing ability test (MAT) was validated [18]. Approval was obtained from the Medical Ethics Committee of the University Medical Center Utrecht (file no. NL12006.041.06). All participants received a written and verbal explanation of the study. Written informed consent was obtained from each participating subject before starting the study.

The alloplastic TJR TMJ involves the reconstruction of both the mandibular condyle and temporal bone fossa, such as a ball and socket similar to the hip prosthesis. This includes a resection of the diseased joint, detachment of several masticatory muscles, and replacement with one of these alloplastic TMJ devices [22,23]. Indications for an alloplastic TJR TMJ combined with one of the end-stage pathologies are ongoing intermittent pain, more than 5 cm on a visual analog scale (VAS) (no pain = 0 cm, severe pain = 10 cm), a restricted mouth opening (<35 mm), low dietary scores (VAS < 5 cm, liquid score = 0 cm, full diet score = 10 cm), and/or occlusal collapse [8]. For the alloplastic TJR TMJ, the Stock Biomet microfixation system (Jacksonville, FL, USA) was used in patients treated in the Jeroen Bosch Hospital. The surgical approach in each patient was through a preauricular and submandibular/retromandibular incision. Both the native mandibular condyle and the coronoid process were removed with loss of attachment of the temporal and lateral pterygoid muscle. The masseter muscle was stripped at its aponeurotic insertion and reconstructed again at closing. The alloplastic fossa component was attached to the zygomatic arch, and the mandibular component was attached to the ramus of the mandible. After the reconstruction was completed, patients were allowed to function immediately. This included the freedom to choose any diet.

### 2.1. Measurements

Age, gender, dental state, preoperative variables (TMJ diagnosis, number of prior TMJ surgeries), operative variables (number, side, time since surgery), and postoperative reports, including postsurgical complications, were collected and taken from the clinical records of the included patients. Masticatory performance, MVBF, active MMO (aMMO), passive MMO (pMMO), pain, patient satisfaction related to their alloplastic TJR TMJ, and masticatory ability were measured in these patients. Of the healthy individuals in this study, age, gender, dental state, masticatory performance, MVBF, and aMMO were collected.

For the objective measurements, the participants were asked to keep their buttocks and lower back against the back of the chair and their knees flexed in a 90-degree flexion, with their feet flat on the floor and no armrest. Their head was kept in a neutral position; the head was considered neutral if the tragus of the ear was in line with the shoulder. To maintain this head position, the participants were asked to focus on a point directly in front of them.

### 2.2. Masticatory Performance

Masticatory performance was measured by the mixing ability test (MAT). The MAT is a valid and reliable test for masticatory performance and is highly suitable when masticatory performance is compromised [16,17,18,19,20]. This test evaluates the capacity of the participants to mix and knead a food bolus after a fixed number of chewing cycles. The tablet consists of two layers of red and blue wax (plasticine modeling wax, nontoxic DIN EN-71, art. no. crimson 52801 and blue 52809, Stockmar, Kalten Kirchen, Germany). The diameter of the tablet is 20 mm. Both layers of red and blue wax are 3 mm thick. When the tablet is chewed on, the colors mix; when the tablet is not chewed on, the colors do not mix, and the intensity of both colors is maximal. The measure of mixing is the spread of the color intensities of both sides.

The wax tablets were offered at room temperature (20°). Each participant masticated twenty times on the tablet. After the participants masticated twenty times on the tablet, the tablet was washed and dried. The chewed tablets were flattened, and both sides were photographed using a high-quality scanner (Epson V750, Long Beach, CA, USA). After that, the spread of the color intensity was measured by computer analysis of the digital images using Photoshop CS3 (Adobe, San Jose, CA, USA). The spread of the color intensities of both images was used as a measure of mixing. This is termed the mixing ability index (MAI). A lower MAI score represents a better-mixed tablet and, thus, better masticatory performance.

### 2.3. Maximum Voluntary Bite Force

MVBF was measured with the unilateral strain-gauge bite force transducer. It has been documented that both unilateral and bilateral bite force transducers can be used as valid and reliable measures [24,25]. The reliability of the results has been reported to depend on the position of the transducer within the dental arch. It has been documented that MVBF recorded posteriorly, both unilateral and bilateral, were notably higher than those recorded anteriorly [26]. Therefore, in this study, MVBF was recorded with the strain-gauge mouthpiece placed on the first molar region. The design consists of a unilateral strain gauge mounted on a mouthpiece. It has a surface area of 100 mm^2^ and a vertical height of 2.8 mm.

To build confidence, the participants were allowed to become familiar with the force transducer by producing several test bites without producing their maximum force. The strain-gauge mouthpiece was placed on the first molar region. The mouthpiece was protected from humidity with a plastic film. After several test bites, the participants were asked to bite as hard as possible for a few seconds. The participants clenched twice on the left side of the jaw and twice on the right side of the jaw with an interval of 10 s in between. The mean of the highest bite force of the left side and the highest bite force of the right side was the MVBF used in this study.

### 2.4. Maximum Mouth Opening

To measure aMMO, participants who routinely used a dental prosthesis were instructed to wear their prosthesis during the measurements. Then, participants were asked to open their mouth as wide as possible to measure aMMO. In patients with an alloplastic TMJ TJR, at first, the overbite of participants was measured. After that, these patients were asked to open their mouth as wide as possible whilst maintaining the head position, and then the distance between the interincisors was measured from the upper right central incisor to the lower right central incisor.

To measure pMMO in patients, the examiner gave a slight overpressure on the edges of the upper and lower front teeth. Then, the distance between the interincisors was measured from the upper right central incisor to the lower right central incisor. The interincisal distance plus the vertical overbite was used in this study to determine the MMO.

In healthy individuals, aMMO was measured extraorally [27]. Two fixed points were marked with a pencil; one point was on the lower side of the chin, and the other was on the tip of the nose. The distance between the two points was measured using a digital slide gauge with the mouth at rest and at its maximum open position. For the resting position measurement, patients were instructed to close their mouth without their teeth making contact. For the maximum open position measurement, patients were instructed to open their mouth as wide as possible.

Each MMO measurement in patients and healthy individuals was performed twice, and the highest outcome was used in the subsequent analyses. Both ways of measuring MMO are reliable and do not differ in their result [28].

### 2.5. Masticatory Ability

To evaluate patients’ masticatory ability, the Dutch version of the Mandibular Function Impairment Questionnaire (MFIQ) was used [29]. This PRO of mandibular function focuses on limitations in chewing various foods, drinking, yawning, impairment of normal activities, and speech. The Dutch MFIQ has 17 items assessing perceived difficulties in mandibular functioning; each item presents a 5-point Likert scale on which patients can indicate the experienced level of difficulty while performing particular mandibular movements or tasks (e.g., speech, daily activities, drinking, laughing, yawning, eating different types of food). The scores are: 0 = no difficulty, 1 = a little difficulty, 2 = quite a bit of difficulty, 3 = much difficulty, and 4 = very difficult or impossible without help. A total score ranging from 0 to 68 is possible, where 0 indicates no mandibular function impairment and 68 a poor functional outcome and very great difficulty.

### 2.6. Pain

Pain was in this study measured by a visual analog scale (VAS_pain_) [30,31] consisting of a horizontal line, with ‘no pain’ (0 cm) on the left end, and on the right end ‘worst pain imaginable’ (10 cm), indicating no pain (0–0.4 cm), mild pain (0.5–4.4 cm), moderate pain (4.5–7.4 cm), and severe pain (7.5–10 cm) [32]. Patients were asked to fill in a VAS_pain_ related to perceived pain during the last week before further measurements took place. Patients also filled in a VAS_pain_ after each measurement (i.e., MAT, MVBF, aMMO, and pMMO).

### 2.7. Patient Satisfaction

VAS_satisfaction_ was used to measure present satisfaction with the alloplastic TJR TMJ [33] consisting of a horizontal line, with ‘no satisfaction’ (0 cm) on the left end and on the right end, ‘extreme satisfaction’ (10 cm).

### 2.8. Statistical Analysis

The presentation of results is primarily descriptive, with frequency and percentages for categorical data, means and standard deviations (SDs) for continuous data, and medians and interquartile ranges (IQRs) for ordinal and non-normally distributed continuous data.

Differences between the alloplastic TMJ TJR group and the healthy group were analyzed by Fisher’s exact test for categorical data, the independent t-test for continuous data, and the Mann–Whitney U test when continuous data were non-normally distributed. Statistical analyses were regarded as significant if the p-value was equal to or lower than 0.05. Data were evaluated using SPSS (IBM version 27.0).

## 3. Results

In total, 15 patients were treated with an alloplastic TMJ TJR in the period from January 2013 to April 2018 at the Jeroen Bosch Hospital. However, one patient was in hospital for a different disease, one patient was not able to speak or understand Dutch or English, and one patient was not willing to participate. Therefore, 12 patients (10 women) were included in this study after TMJ TJR with a unilateral Biomet Microfixation stock prosthesis (Inc., Jacksonville, FL, USA). The mean age at the time of the measurements was 61.69 years (±6.77), and the mean time after surgery on the day the measurements took place was 2.54 years (±1.26). Four patients had their natural teeth, two patients had implant-retained overdentures in the upper and lower jaw, and three patients had complete dentures (Table 2). The patients had an MAI of 19.56 (±2.45), an MVBF of 201.04 (±159.72), an aMMO of 42.50 (±7.93), a pMMO of 44.50 (±6.72), and an MFIQ outcome of 33.42 (±15.72), as depicted in Table 3.

The Shapiro–Wilk test showed that all VAS_pain_ and VAS_satisfaction_ outcomes in patients were not normally distributed. VAS_pain_ during last week, pain during MAT, pain during MVBF, and pain during aMMO had a median of 0.00, indicating that the patients had no pain. Patients’ pain during pMMO had a median of 1.75, indicating mild pain. Patients were very satisfied with their TMJ TJR (Table 3).

A total of 12 matched healthy individuals (10 women) aged 60 (±6.0) years were included in this study. Four healthy individuals had their natural teeth, two healthy individuals had implant-retained overdentures in the lower jaw and natural teeth in the upper jaw, and three healthy individuals had complete dentures (Table 2). The healthy individuals had an MAI of 18.21 (±1.89), MVBF of 463.88 (±301.16), and aMMO of 51.79 (±6.96), as depicted in Table 3.

**Figure 1:**
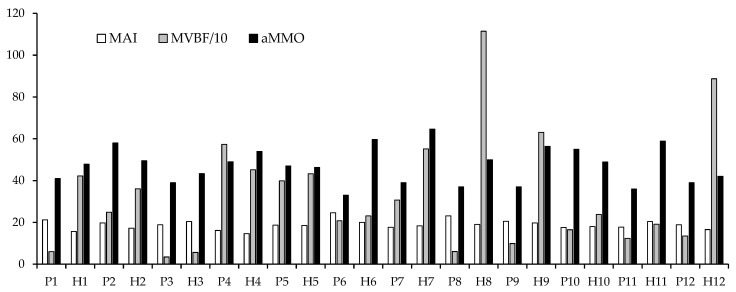
Individual outcomes of patients and healthy individuals: P1, patient 1 of Table 2; H2, healthy individual 2 of Table 2; MAI, mixing ability index; MVBF/10, maximum voluntary bite force divided by 10; aMMO: active maximum mouth opening.

Statistical comparison of patients with an alloplastic TMJ TJR and healthy individuals showed that patients with an alloplastic TMJ TJR had a significantly lower MVBF (*p* = 0.014) and aMMO (*p* = 0.006) compared with healthy individuals. There was no significant difference in MAI (*p* = 0.145) between patients with an alloplastic TMJ TJR and healthy individuals. Individual outcomes for MAI, MVBF, and aMMO can be found in Figure 1 for both patients with an alloplastic TMJ TJR and healthy individuals.

## 4. Discussion

The masticatory performance in patients with a TJR TMJ showed to be equal to the healthy individuals matched on age and dental state. However, maximum voluntary bite force and active maximum mouth opening were significantly lower. Looking at MFIQ masticatory outcomes, patients with a TJR TMJ experienced limitations with eating hard and tough food and biting something big; however, these deficits were still very little (median: 0.5–1.5). These masticatory ability outcomes are comparable with the masticatory ability found in patients with a TJR TMJ in two other observational studies [34,35].

In this study, MVBF was significantly lower in patients with TMJ TJR than in healthy individuals. A possible explanation could be that removal of the temporal and lateral pterygoid muscle leads to a loss in muscle action and neuromuscular control. Moreover, the masseter muscle is stripped at its aponeurotic insertion; even though this muscle is reconstructed at closing, it still has an impact on power and neuromuscular control [13,36]. An additional explanation could be the lack of a postoperative rehabilitation protocol. The patients in this study had no physiotherapeutic support after the replacement of an alloplastic TJR TMJ. However, it has been reported in the literature that functions are not optimized after the replacement of an alloplastic TJR TMJ when patients do not follow a rehabilitation protocol [37]. While there is growing interest in this surgical intervention, there is still a paucity of data about postoperative protocols for physiotherapy and functional rehabilitation in patients with an alloplastic TJR TMJ. It has been stated that by the year 2030, there will be an increasing demand for the use of TJR TMJ prostheses in the United States of America [38]. In addition, in Europe, there is an increasing demand for the use of TJR TMJ prostheses [39]. Future research into the influence of rehabilitation interventions in patients with an alloplastic TJR TMJ, such as orofacial physiotherapy, is crucial since important goals of this surgery include functional improvement in the masticatory system [13]. Furthermore, we observed great interindividual differences in MVBF. In a prospective study, substantial interindividual differences were also observed in MVBF in patients with an alloplastic TJR TMJ [13]. An attempt to explain this wide interindividual variance is the voluntary influence of the measurement. The willingness and courage to bite as hard as possible are known to be influenced by the mental attitude of the patient and also by the comfort of the patient’s teeth [1,14].

aMMO was also significantly lower in patients with a TJR TMJ than in healthy individuals. Patients with a TJR TMJ experienced only a little restriction with yawning. Thereby, the patient results showed an aMMO ranging from 33 to 58 mm and a pMMO ranging from 35 to 59 mm, which demonstrates that these patients had a satisfactory mandibular range of motion. In most other studies, lower MMO results were found postoperatively [35,40,41,42,43,44,45,46,47,48,49,50,51,52]. Only one prospective cohort study showed similar MMO results at the 12-month follow-up and thereafter [53]. This difference cannot be explained by the use of the Biomet prosthesis because the MMO results of other studies using the Biomet found lower as well as equal MMO results [35,43,51,53].

Our study demonstrated that pain scores in most patients were very low. These findings are consistent with the findings of previous studies [15,47,54]. Another interesting observation is that in our study, patients reported being extremely satisfied with their alloplastic TJR TMJ. In previous studies, patients also reported being satisfied with their TJR TMJ, but were, on average, not as highly satisfied compared with the patient-reported outcomes in our study [55,56]. In an 8-year longitudinal follow-up study, it was reported that patient satisfaction was positive even when pain and poor mandibular function were reported in these patients. They assumed that the effort made to inform patients what to expect had contributed to treatment satisfaction [56]. In our study, one patient reported experiencing moderate pain during all measurements and during the last week. This patient also scored the lowest on the satisfaction rating scale. Therefore, in our study, the patients were extremely satisfied even when MVBF was impaired because they reported experiencing almost no pain, and the most important reason patients first seek treatment is TMJ pain [57].

The strength of this study is that, to our knowledge, no other studies have reported on masticatory performance in patients treated with an alloplastic TJR TMJ. Therefore, this study may add knowledge about masticatory functioning in patients with a TJR TMJ. Thereby, this study contributes to a better understanding of the results after replacing the temporomandibular joint. However, limitations also need to be taken into consideration. A limitation of this study is the limited number of participants. In addition, the findings are based on postoperative measurements and cannot be compared with preoperative findings. Despite these limitations, the findings of this study can provide a basis for future research into measuring the masticatory functioning outcomes of this surgical option in a larger number of patients.

In conclusion, this study revealed that most patients with an alloplastic TJR TMJ were able to function without pain, showed good masticatory performance, and were highly satisfied with their alloplastic TJR TMJ. However, MVBF and aMMO were lower than in healthy individuals. Despite the fact that the success of alloplastic TJR TMJ has been established in the literature, research has to be continued to optimize rehabilitation care in patients with an alloplastic TJR TMJ.

## Figures and Tables

**Table 1:** In- and exclusion criteria for patients treated with a TMJ TJR.

Inclusion Criteria	Exclusion Criteria
Unilateral or bilateral stock total TMJ reconstruction	Edentulous and had no denture prosthesis
Minimal 3 months postsurgery	TJR TMJ surgery-related complication
	Not able to speak or understand Dutch or English

TMJ: temporomandibular joint; TJR: temporomandibular joint replacement. Surgical and postsurgical procedures.

**Table 2:** Demographic and clinical characteristics of patients treated with a TMJ TJR and matched healthy individuals.

Patient Number	Sex	Age at Measurement Moment (Years)	Time after Surgery (Years)	Dental State	Diagnoses	TJR Side	Prior TMJ Surgery at TJR Side	Healthy Individual Number	Sex	Age at Measurement Moment (Years)	Dental State
1	Female	59.79	2.30	Natural dentition	Arthrosis	Left	0	1	Female	58.35	Natural dentition
2	Male	70.34	2.66	Implant-retained overdenture in upper and lower jaw	Arthrosis	Right	1	2	Male	68.98	Implant-retained overdenture in lower jaw and natural dentition in upper jaw
3	Female	71.90	2.12	Complete denture	Arthrosis	Left	0	3	Female	61.74	Complete denture
4	Female	59.76	2.43	Natural dentition	Condylar resorption	Right	1	4	Female	55.96	Natural dentition
5	Female	60.58	2.84	Implant-retained overdenture in upper and lower jaw	Ankyloses	Right	0	5	Female	60.03	Implant-retained overdenture in lower jaw and natural dentition in upper jaw
6	Female	56.96	0.52	Complete denture	Osteoarthritis	Left	0	6	Female	56.62	Complete denture
7	Female	50.68	3.63	Natural dentition	Arthrosis	Right	1	7	Female	50.56	Natural dentition
8	Female	51.12	3.82	Natural dentition	Arthrosis	Right	1	8	Female	50.89	Natural dentition
9	Female	67.18	0.39	Natural dentition	Osteoarthritis	Right	1	9	Female	64.66	Natural dentition
10	Male	66.55	4.27	Complete denture	Osteoarthritis	Left	0	10	Male	69.05	Complete denture
11	Female	64.37	1.68	Complete denture	Osteoarthritis	Left	1	11	Female	61.48	Complete denture
12	Female	61.01	3.86	Natural dentition	Ankyloses	Right	0	12	Female	61.83	Natural dentition

TMJ: temporomandibular joint; TJR, temporomandibular joint replacement.

**Table 3:** Comparison of characteristics and outcomes of patients treated with an alloplastic total temporomandibular joint prostheses and healthy individuals.

	Patients with TMJ TJR N = 12)	Healthy Individuals (N = 12)	*p*-Value
Sex (N (%))			1.000 ^‡^
Female	10 (83%)	10 (83%)	
Male	2 (17%)	2 (17%)	
Age (years) (mean (SD))	61.69 (6.77)	60.01 (6.00)	0.528 ^†^
Time after surgery (mean (SD))	2.54 (1.26)	-	-
Prior TMJ surgery at TJR side			-
Yes	6 (50%)	-	
No	6 (50%)	-	
Dental state (N (%))			0.456 ^‡^
Natural dentition	6 (50%)	6 (50%)	
Implant-retained overdenture in lower jaw and natural dentition in upper jaw	0 (0%)	2 (17%)	
Implant-retained overdenture in lower and upper jaw	2 (17%)	0 (0%)	
Complete denture	4 (33%)	4 (33%)	
Mixing ability index (mean (SD))	19.56 (2.45)	18.21 (1.89)	0.145 ^†^
Maximum voluntary bite force (mean (SD))	201.04 (159.7)	463.88 (301.16)	0.014 *^†^
Active maximum mouth opening (mean (SD))	42.50 (7.93)	51.79 (6.96)	0.006 **^†^
Passive maximum mouth opening (mean (SD))	44.50 (6.72)	-	-
Pain during last week (median (IQR))	0.00 (0.00–0.23)	-	-
Pain during mastication of Mixing Ability Test (median (IQR))	0.00 (0.00–0.30)	-	-
Pain during maximum bite force at the non-operative site (median (IQR))	0.00 (0.00–0.00)	-	-
Pain during maximum bite force at the operative site (median (IQR))	0.00 (0.00–2.85)	-	-
Pain during active maximum mouth opening (median (IQR))	0.00 (0.00–3.53)	-	-
Pain during passive maximum mouth opening (median (IQR))	1.75 (0.00–3.53)	-	-
Satisfaction (median (IQR))	10.00 (9.80–10.00)	-	-
Mandibular Functional Index Questionnaire; total (mean (SD))	33.42 (15.72)	-	-
Social activities (median (IQR))	0.00 (0.00–1.00)	-	-
2. Speaking (median (IQR))	0.00 (0.00–1.00)	-	-
3. Biting something big (median (IQR))	1.00 (0.00–3.75)	-	-
4. Eating hard food (median (IQR))	1.00 (0.00–2.00)	-	-
5. Eating soft food (median (IQR))	0.00 (0.00–0.00)	-	-
6. Daily activities (median (IQR))	0.00 (0.00–0.75)	-	-
7. Drinking (median (IQR))	0.00 (0.00–0.00)	-	-
8. Laughing (median (IQR))	0.00 (0.00–0.00)	-	-
9. Chewing resistant food (median (IQR))	1.50 (0.00–3.75)	-	-
10. Yawning (median (IQR))	1.00 (0.00–1.75)	-	-
11. Kissing (median (IQR))	0.00 (0.00–0.00)	-	-
12. Eating hard cookies (median (IQR))	0.50 (0.00–1.00)	-	-
13. Eating meat (median (IQR))	0.50 (0.00–1.00)	-	-
14. Eating raw carrot (median (IQR))	1.50 (0.00–4.00)	-	-
15. Eating French bread (median (IQR))	0.50 (0.00–2.75)	-	-
16. Eating peanuts (median (IQR))	0.50 (0.00–3.75)	-	-
17. Eating whole apple (median (IQR))	1.50 (0.00–4.00)	-	-

*, *p* < 0.05; **, *p* < 0.01; ‡, Fisher’s exact test; †, independent *t*-test; IQR, interquartile range; SD, standard deviation; TMJ, temporomandibular joint; TJR, temporomandibular joint replacement.

## Data Availability

The data in this study are available on request.
